# Protective Effects and Benefits of Olive Oil and Its Extracts on Women’s Health

**DOI:** 10.3390/nu13124279

**Published:** 2021-11-27

**Authors:** Thanh Truong Giang Ly, Jisoo Yun, Dong-Hyung Lee, Joo-Seop Chung, Sang-Mo Kwon

**Affiliations:** 1 Laboratory for Vascular Medicine and Stem Cell Biology, Department of Physiology, Medical Research Institute, School of Medicine, Pusan National University, Yangsan 50612, Korea; lythanhtruonggiang@gmail.com (T.T.G.L.); jsyun14@hanmail.net (J.Y.); 2 Convergence Stem Cell Research Center, Pusan National University, Yangsan 50612, Korea; 3 Department of Obstetrics and Gynecology, Pusan National University Yangsan Hospital, Yangsan 50612, Korea; ldh0707@hanmail.net; 4 Department of Hematology-Oncology, Medical Research Institute, Pusan National University Hospital, Busan 49241, Korea

**Keywords:** olive oil, mediterranean diet, oleuropein, hydroxytyrosol, breast cancer, gynecologic cancer, osteoporosis, postmenopausal disorders

## Abstract

Women and men share similar diseases; however, women have unique issues, including gynecologic diseases and diseases related to menstruation, menopause, and post menopause. In recent decades, scientists paid more attention to natural products and their derivatives because of their good tolerability and effectiveness in disease prevention and treatment. Olive oil is an essential component in the Mediterranean diet, a diet well known for its protective impact on human well-being. Investigation of the active components in olive oil, such as oleuropein and hydroxytyrosol, showed positive effects in various diseases. Their effects have been clarified in many suggested mechanisms and have shown promising results in animal and human studies, especially in breast cancer, ovarian cancer, postmenopausal osteoporosis, and other disorders. This review summarizes the current evidence of the role of olives and olive polyphenols in women’s health issues and their potential implications in the treatment and prevention of health problems in women.

## 1. Introduction

*Olea europaea*, (Oleaceae) which is commonly known as olive tree, is one of the oldest species of trees in the Mediterranean region. Olive oil (OO) is extracted from olives—the fruits of the olive tree. The crucial role of OO was investigated in the 7th century BC. Some ancient scientists have recommended using OO in several diseases related to the stomach and skin [1]. While Mediterranean countries produce about 70% of the OO in the world, Australia and USA also produce significant amounts of OO. However, the variety and quality of OO differ among these countries [2].

One of the most prominent parts of the Mediterranean diet (MD) is OO consumption, which is the principal source of fats. Other components of the MD include frequent consumption of various vegetables and fruits, cereals, fish and seafood, moderate alcohol intake, and relatively low meat intake. Current data on MD suggest that OO and its components have shown preventive effects in cancer [3,4], cardiovascular diseases [3,4], diabetes [3,4], and other diseases [4,5]. Monounsaturated fatty acid (oleic acid) and polyphenol constituents (such as oleuropein, hydroxytyrosol, and tyrosol) were important components that explain the protective role of OO in these diseases [6,7,8]. Among the phenolic components of OO, oleuropein (OLP) is considered the most effective biomolecule [9,10].

Even though there is similarity in diseases among men and women, women have specific issues related to reproductive characteristics, including menstruation, menopause, and post menopause, with a large range of disorders that manifest during this period, as well as gynecological diseases. This review focusses on these women-specific conditions.

Disease prevention and potential treatment therapy research are critical requirements in medical science. In the recent decades, scientists have paid more attention to natural products and their derivatives in order to investigate their effects on disease prevention and treatment. OO and its active components are potential agents with promising research results. Almost all human clinical trials have evaluated the beneficial effects of OO in the context of MD. Therefore, the role of OO requires further investigation.

Therefore, herein, we collected current data from cellular, animal, and human studies regarding the role of MD and OO and its components in various aspects of women’s health. The studies that were used in this review included in vitro studies, in vivo studies, meta-analyses, randomized controlled trials, and clinical trials. Therefore, this review suggests possible future research directions in this area.

## 2. Structure and Bioactivity

### 2.1. OO Subtypes

As described by the International Olive Council (IOC), virgin olive oils are the oils obtained from the fruit of the olive tree (*Olea europaea* L.) solely by mechanical or other physical means under conditions, particularly thermal conditions, that do not lead to alterations in the oil, and which have not undergone any treatment other than washing, decantation, centrifugation, and filtration. In addition, sensorial and chemical properties determine the classification: Extra virgin olive oil (EVOO) is a virgin olive oil which has a free acidity, expressed as oleic acid, of not more than 0.8 g per 100 g, and the other characteristics of which correspond to those fixed for this category in the IOC standard. Virgin olive oil (VOO) which has a free acidity, expressed as oleic acid, of not more than 2 g per 100 g and the other characteristics of which correspond to those fixed for this category in the IOC standard. Refined olive oil is the olive oil obtained from virgin olive oils by refining methods that do not lead to alterations in the initial glycerides’ structure. It has a free acidity, expressed as oleic acid, of not more than 0.3 g per 100 g and its other characteristics correspond to those fixed for this category in the IOC standard. When we talk about Olive oil in general (OO) we considered that is the oil consisting of a blend of refined olive oil and virgin olive oils fit for consumption as they are. It has a free acidity, expressed as oleic acid, of not more than 1 g per 100 g and its other characteristics correspond to those fixed for this category in the IOC standard [11]. EVOO has the best organoleptic characteristics [4]. EVOO and OO had nearly the same fatty acid component but very different phenolic content [12]. EVOO contains the highest concentration of polyphenols [13]. EVOO has a flying flavor and light color due to fatty acid removal [14]. Many factors that influenced the quality of OO include pre-harvest factors (the cultivar, growing area, environmental condition, soil, tree age, treatment, irrigation, fruit ripening, harvest time, fruit picking) and post-harvest factors (fruit storage, leaves removing and washing, fruit crushing, paste malaxation, oil extraction systems, oil storage, cooking) [15]. OO constituents can be divided into the saponifiable fraction (98.5–99.5%) and the unsaponifiable fraction (0.5–1.5%) [4]. Triglycerides are the most important part of the saponifiable fraction. Unsaponifiable fraction contains hydrocarbons, chlorophylls, tocopherols, aliphatic alcohols, sterols, phenolic compounds, volatile compounds [4]. Oleic acid is the major monounsaturated fatty acid in OO, accounting for approximately 83% [16]. In EVOO, the mean concentration of total phenolic content was 483 mg·kg^−1^ measured by qNMR, although the phenolic content registered a large variation among the various cultivars [17]. Triacylglycerol content depends on the cultivar and the ripening stage [18]. Microclimatic, agronomic, oil’s extraction conditions, the cultivar, and the harvest date influenced the sterols [19], fatty alcohols [20,21], and waxes [22,23]. Phenolic and fatty acid composition is influenced by harvest date [24] and growth environment [25].

**Figure 1:**
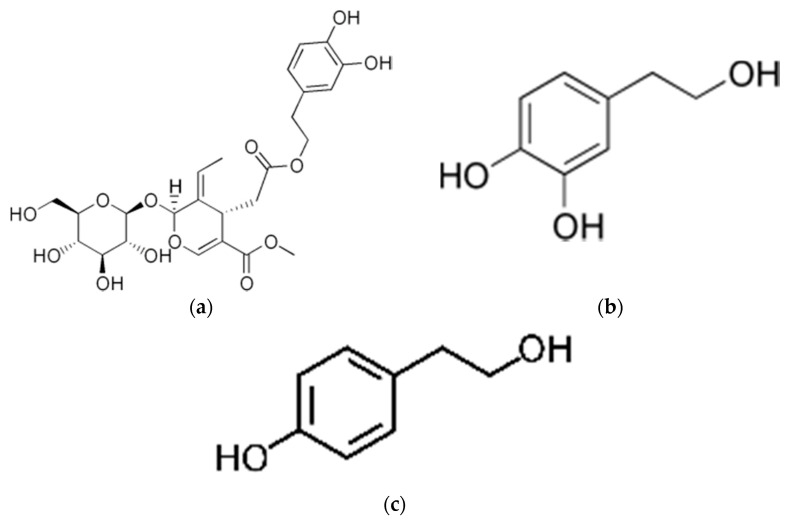
Chemical structure of (**a**) oleuropein, (**b**) hydroxytyrosol, and (**c**) tyrosol.

OO polyphenols include tyrosol (4-hydroxyphenylethanol), hydroxytyrosol (3,4-dihydroxyphenylethanol), oleuropein, caffeic acid, vanillic acid, syringic acid, p-coumaric acid, o-coumaric acid, protocatechuic acid, 4-hydroxybenzoic acid, 4-hydroxyphenylacetic acid and 3,4-dihydroxyphenylacetic acid [26]. The chemical structure of representative phenols was illustrated in Figure 1. Oleocanthal [27], tyrosol, HT [28], and OLP [29] have a wide variety of beneficial health effects [30].

### 2.2. Bioactivities

OO extracts have shown protective effects against several diseases, such as hypertension, diabetes, sepsis, obesity, osteoporosis, neurodegeneration, and chronic kidney diseases [31,32,33]. OO consumption decreases the risk of all-cause mortality [34]. OO and its active derivatives showed antioxidant and anti-inflammatory effects [35]. Moreover, OO has antibacterial properties [36].

MD is associated with a risk reduction in the incidence and mortality of many types of cancers [37,38,39]. Trichopoulou et al. crudely calculated that in the group eating a traditional healthy MD diet, there was a 25% lower incidence of colorectal cancer, 15% lower incidence of breast cancer, and 10% lower incidence of prostate, pancreas, and endometrial cancer compared to the Western diet group [38]. MD can reduce the inflammatory process that contributes to cancer pathogenesis [40,41]. MD maintains the gut microbiota balance, which reduces inflammation in the intestinal mucosa, resulting in cancer reduction [42,43]. Polyphenols also show anti-cancer effects through various mechanisms related to apoptosis, proliferation, inflammation, angiogenesis, and cell cycle arrest [44]. HT showed a protective effect in the aging process via AMP-activated protein kinase (AMPK) and autophagy [33]. OO consumption decreased the risk of stomach cancer, ovarian cancer, colon cancer, endometrium cancer, particularly breast cancer. These beneficial findings have been reported in several meta-analysis studies [45,46,47]. OO, most likely oleic acid, regulates the *HER2* gene associated with cancer [48]. Bioactivities of OO were characterized by a high level of monounsaturated fatty acid and antioxidant effects of polyphenols. Although various studies showed the protective effects of OO in the prevalence of several types of cancers. However, the mechanism by which the effects of OO reduce the risk of cancer remains poorly understood. So, it requires more studies that focus on the mechanism of how OO and its bioactive components impact the development of cancer, such as the regulation of the expression of the oncogenes.

## 3. Cancer in Women

Cancer remains a major cause of death in humans. In 2020, the cancer statistics calculated by Ferlay et al. included 19.3 million new cases and almost 10 million cancer-related deaths. Breast cancer is the most common cancer worldwide, with 2.26 million cases [49]. Cancer negatively affects various aspects of life, such as the economy and society as well as health and wellbeing. Although there have been therapeutic advances, including the development of targeted therapies, cancer patients still face short-term and long-term side effects from current therapies and medication resistance. More studies are required to investigate potential low-risk therapies for cancer prevention and treatment to improve the outcome and quality of life of cancer patients. Natural products have recently attracted attention for their anticancer role as potential adjunctive therapies due to their effects and because they are well tolerated. OO extracts and their bioactive components are some of the agents that have been investigated. 

### 3.1. Ovarian Cancer

Ovarian cancer is one of the most common gynecologic cancers in both developed and developing countries, negatively affecting women’s health and fertility. Primary epithelial ovarian cancer is the most common type of ovarian cancer. Risk factors for ovarian cancer include increasing age, infertility, and endometriosis. Approximately 20% of ovarian cancer cases have familial factors. Ovarian malignancy is diagnosed at an average age of 63. Major therapies for ovarian cancer include surgery and chemotherapy. Ovarian cancer patients with advanced stage disease face a high risk of relapse and poor outcomes [50,51]. 

In 2021, Benot-Dominguez et al. reported that olive leaf extract (OLE) reduces the cell proliferation cell cycle and increases apoptosis via mitochondrial impairment, which leads to a decrease in tumor growth [52]. Shabani suggested that OLP induces apoptosis, inhibits cell proliferation, and decreases cisplatin resistance by regulating miRNA expression [53]. Although older radiation therapy is rarely used in ovarian cancer, the improved radiotherapy techniques showed potential effects in ovarian cancer treatment [54]. OLP increases the sensitivity to radiotherapy in ovarian cancer patients [55]. Polyomavirus enhancer activator 3 (PEA3) a transcription factor of ETS family [56], PEA3 contributed to the organs forming include kidney [57], mammary gland [58], and limb buds [59]. PEA3 inhibits tumor formation that depends on HER-2/neu [60,61]. Menendez et al. suggested a protective mechanism of oleic acid in cancer via inhibition of the HER-2/neu gene promoter, which depends on PEA3 [62]. Tzonou et al. showed that there was a statistically significant inverse association between mono-unsaturated fat (mostly OO) consumption and ovarian cancer in a case-control study in Greece [63]. Bosetti et al. reported similar results [39,64]. However, a review of meta-analyses of observational studies and randomized trials showed that the association between ovarian cancer and the MD remains elusive [32].

### 3.2. Breast Cancer

Breast cancer is the leading cause of cancer-related deaths in women. Genetic factors contribute to the risk of breast cancer [65]. Currently, breast cancer treatment therapies include surgery, radiation, endocrine therapy, neoadjuvant chemotherapy, and biological therapy. The five-year survival rate of breast cancer in women is approximately 90% in the United States [66]. The choice of treatment therapies depends on the types of breast cancer, including triple-negative breast cancer, HER2 (human epidermal growth factor receptor 2)-negative cancer, and hormone receptor (HR)-positive breast cancer, and HER1-positive diseases [67]. The application of adjuvant systemic therapy reduces mortality in breast cancer [68,69,70].

The anticancer role of OO extracts and their bioactive components in breast cancer has been evaluated in numerous in vitro, in vivo, and several clinical trials [71,72,73,74,75].

The OO extract contains several types of compounds. OLP has been shown to play the most important role in breast cancer cell toxicity [76,77]. In breast cancer, OLP inhibits cell proliferation, induces apoptosis, and induces cell cycle arrest [78,79,80,81,82]. Several mechanisms have been suggested, including miRNA dysregulation [79]. According to Bent-Dominguez, OLE, whose main compound is OLP, was found to increase reactive oxygen species (ROS) generation results in cell cycle delay, apoptosis, and mitochondria dysfunction [52]. Another study showed that phenolic extracts induced cell death and increased ROS production [80]. Hassan supported that OLP-induced apoptosis due to p53 pathway activation is regulated by the *BAX* and *BCL2* genes [83]. Biosynthesized OLP aglycone (OLA) inhibited tamoxifen-resistant MCF-7 cell growth, whereas normal breast epithelial cells did not change. OLA also inhibits the cell cycle and induces apoptosis [84]. Messeha et al. showed that OLP altered the mRNA expression related to the apoptosis process of two kinds of triple-negative breast cancer cell lines, MDA-MB-468 and MDA-MB-231, and supported that OLP is more effective in MDA-MB-468 than in MDA-MB-231 [82]. The effect of OLP was higher in MDA-MB-231 cells than in MCF-7 cells. OLP reduces breast cancer cell growth by regulating the cell cycle by decreasing NF-κB and cyclin D1 expression and increasing p21 expression [85]. Epithelial-mesenchymal transition (EMT) is a fundamental step in the metastasis process [86,87]. In 2019, Choupani et al. showed that OLP inhibits EMT via downregulation of sirtuin1 leads to inhibition of breast cancer cells migration [88]. In addition, combination therapy with doxorubicin and OLP may be possible due to their synergistic effect on apoptosis of human breast cancer cells [88].

HT, the main phenolic compound of the olive oil, has also been shown to be effective in breast cancer. It inhibits cell growth and cell cycle arrest by reducing the expression of cyclinD1 by upregulating c-Jun and reducing pin-1 expression [89]. Moreover, OLP and HT decrease the migration and invasion of estrogen-positive breast cancer cell lines such as MCF7 [81,90] and T47D via autophagy activation [90] or histone deacetylase regulation [81,91]. Sirianni et al. showed that OLE and HT inhibit the ERK1/2 activation that is dependent on E2 [92].

Another bioactive phenolic compound from EVOO purification is S-(-) oleocanthal (OC). OC inhibited triple-negative breast cancer progression and metastasis to the lung in two heterogeneous triple-negative breast cancer animal models, and no considerable toxicity was observed. Additionally, using a microarray gene signature, this study showed that OC treatment protects almost all steps of cancer progression, including cell-to-cell adhesion signaling, interaction, invasion, and migration [93].

OO and its active components demonstrated effects in cancer formation, progression, metastasis, prognosis, and response to treatment therapy. MCF-7 breast cancer cell line proliferation requires the protein tyrosine phosphatase 1B (PTP1B) [94], an enzyme that plays a crucial anti-cancer role [95]. OLP reduces PTP1B activity, which is correlated with cell growth and cell cycle delay. This suggests that PTP1B phosphatase may be a target for OLP treatment in breast cancer [96]. HER-2 plays an important role in various aspects of cancer progression in breast cancer, including its etiology, progression, and response to therapies. Over-expression of HER2 leads to poor prognosis, decreased relapse time, and low survival [97,98,99]. This study showed that EVOO inhibits HER2 activity by increasing the proteasomal degradation of this protein [100]. Menendez et al. showed that EVOO polyphenols also inhibit fatty acid synthase (FASN) expression in HER-2-overexpression breast cancer [101]. FASN is strongly expressed in many human cancers and is positively correlated with poor prognosis and low survival; therefore, it is considered an oncoprotein [102]. This study also examined the role of EVOO, especially the role of OLP aglycone in the improvement of the effect and resistance of trastuzumab in vitro [77]. Therefore, OO may be synergistic with the current therapies. OLP also showed anti-metastatic effects by decreasing matrix metalloproteinase (MMP) expression and increasing the expression of tissue inhibitors of metalloproteinases [103]. HDCA plays an important role in cell proliferation and apoptosis [104,105]. OLP decreases HDCA expression, including HDAC2, HDAC3, and HDAC4 [81,91]. Plasminogen activator inhibitor-1 (PA-1) contributes to blood clotting, and increased PA-1 expression is associated with poor outcomes in breast cancer [106,107]. Tzekaki et al. supported OLP as a strong binder to PA-1. EVOO and OLP treatment inhibited PA-1 expression in ER-/PR- breast cancer cell lines. Moreover, this study showed that EVOO and OLP suppressed cell growth and caspase activation [108].

Cancer stem cells (CSC), characterized by self-renewal and differentiation, contribute to the pathogenesis of therapy resistance, tumor formation, and metastasis abilities [109,110,111]. Therefore, CSC are considered a target for investigating novel therapies. Corominas-Faja et al. showed EVOO-derived crude phenolic extract (EVOO-PE) inhibited CSC formation in the first step. Because of the most abundant compound in EVOO-PE, purified OLA and decarboxymethylated oleuropein aglycone (DOLA) were used for further experiments. They observed DOLA has greater inhibitory effects compared to OLA. DOLA significantly decreased the mammosphere-forming in four traditional breast cancer cell lines (DCIS.com, T47D, ZR-75-1, and SUM-159). For in vivo tumor formation ability, they used SM-159 cells pre-treated DOLA 20µg/L for 3 days with daily re-feeding and injected subcutaneously. DOLA reduced tumor formation compared to the control group. DOLA also suppressed the growth of tumors in the orthotopic implantation model. DOLA also regulated the gene expression related to stem cell fate. In silico computational studies determined DOLA as a dual mTOR/DNMT inhibitor [112].

In summary, underlying molecular mechanisms of OO function, especially OLP, it has been suggested that diverse signaling pathways related to apoptosis, cell growth, cell cycle, and ROS generation contribute to tumor growth and metastasis. In addition, it regulates many genes related to the prognosis and outcomes of breast cancer patients.

Regarding OO, MD, and its constituents in clinical trials, long-term MD+EVOO reduced breast cancer incidence in a study (*n* = 4152) performed from 2003 to 2009 [74]. In 1208 patients with early stage breast cancer, a MD combined with exercise decreased breast cancer recurrence [75]. Skouroliakou et al. evaluated the MD intervention in postmenopause breast cancer survivors for 6 months. There was a significant decrease in body weight, body fat mass, waist circumference, body mass index, and increase in the vitamin C, CoQ10 levels in the intervention group. In the comparison between the two groups at the end of the study, registered blood glucose concentration was significantly lower while the vitamin C, CoQ10 levels were considerably higher compared to the control group [113].

In 2018, in a clinical trial using HT in combination with omega-3 fatty acid and curcumin, Martinez et al. found reduced levels of C-reactive protein, a marker of inflammation and pain in early stage breast cancer patients treated with hormonal therapy [114].

OO intake can reduce breast cancer risk [45,46,47,115]. In 2021, a meta-analysis that assessed the OO consumption and breast cancer risk data from 10 observational studies (two prospective studies and 8 case-control studies) showed that OO intake may decrease breast cancer risk, the random effects summary OR for breast cancer was 0.48 (95% CI = 0.09–2.70) for prospective studies and 0.76 (95% CI = 0.54–1.06) in case-control studies, comparing women with the highest intake to those with the lowest intake category of olive oil. The relationship between breast cancer risk and dose-response olive oil was not significant; the OR (95% CI) for breast cancer in the dose-response meta-analysis with a 14 g/day increase in olive oil intake was 0.93 (0.83–1.04) [116].

### 3.3. Cervical Cancer

Cervical cancer is the fourth most common cancer in women and is one of the leading causes of death in developing countries [117]. In 2020, 604,000 new cases of cervical cancer and 342,000 deaths were reported worldwide [118]. Human papillomavirus (HPV) infection accounts for 99.7% of cervical cancers [119]. Treatment of cervical cancer includes surgery, chemotherapy, and radiation, which vary with disease stage.

Torics et al. (2020) assessed the effect of the phenolic compounds in EVOO on cervical cancer. They showed that EVOO phenolic extracts inhibit cell growth, although in combination with current cancer therapy such as irinotecan and 5-fluorouracil, the results were not statistical different [120]. OO polyphenols increased GSH levels, the most crucial intracellular antioxidant molecules measured by flow cytometry, but did not alter ROS levels. HT may have a higher antioxidant effect than tyrosol [121]. A cross-sectional study in Italy by Barchitta et al. suggested that MD might lower the risk of HPV infection and high-grade cervical intraepithelial neoplasia [122]. OLP increases apoptosis by upregulating the JNK/SPAK signaling pathway [123].

Another study showed that a high olive diet enhanced cervical cancer growth and metastasis in a mouse xenograft model. Oleic acid increases cell proliferation, migration, and invasion. Oleic acid induces CD36 via SRC/ERK activation, which contributes to cervical cancer formation and the progression of cervical cancer [124]. Zhang et al. reported that a high olive diet can enhance tumor growth in cervical cancer in vivo. Oleic acid increased the proliferation and migration of cervical cancer cells. This study also showed the different gene expression patterns altered by the olive oil diet and a set of hub genes for further investigation [125].

### 3.4. Endometrial Cancer

Endometrial cancer is one of the most common cancers in women. Estrogen is a major risk factor, obesity, low physical activity, and poor nutrition are also other risk factors for endometrial cancer. The major histopathological features of endometrial cancer originate from the epithelium. Its incidence peaks between the ages of 60 and 70 years. Treatment methods include surgery and adjuvant chemotherapy for high-risk endometrial cancer [126,127,128]. The study evaluating the role of OO and its extracts in endometrial cancer is not available.

### 3.5. Vaginal Cancer

The incidence of vaginal cancer is lower than that of ovarian and cervical cancers. Most vaginal tumors are squamous carcinomas, and other histologic types are less common. The majority of vaginal cancers are a result of metastasis from other organs such as the endometrium, cervix, vulva, ovary, breast, rectum, and kidney. The mean age of the patients tends to be around 60 years. Treatment therapy decisions depend on many factors, including the location, size, and clinical stage of the tumor, and these are also prognostic factors for vaginal cancer patients. Treatment therapies include surgery, radiation, and chemotherapy [66,126,129,130]. The evidence related to the association between OO, active phenolic constituents, and vaginal cancer is not available.

### 3.6. Vulvar Cancer

Primary vulvar cancer is a rare disease that is less common than vaginal cancer. The major histopathology is squamous cell carcinoma. Treatment therapy consists of surgery, radiation, and chemotherapy. HPV infection is a major type of vulvar cancer [131,132,133]. The effect or impact of MD or OO and its components in vulvar cancer is not available. A study assessed the risk of fat consumption in mice in relation to reproductive system tumor formation. They used four groups of fat, including corn oil, fish oil, OO, and lard. There were no significant differences among these groups [134].

## 4. Postmenopausal Disorders

Postmenopausal women suffer from several disorders due to the reduction in estrogen and other hormones, including emotional fluctuations, hot flashes, depression, anxiety, and vaginal dryness from perimenopause to post menopause [135]. The incidence of obesity, metabolic syndrome, cardiovascular diseases, and osteoporosis is associated with menopause [136]. In ovariectomized rats, EVOO reduced IL-6, malonyldialdehyde, and nitrate levels. Thus, OO has antioxidant and anti-inflammatory effects during menopause. This study also evaluated cancer markers, including carbohydrate antigen 125 (CA125), carcinoembryonic antigen (CEA), α-fetoprotein (AFP), and carbohydrate antigen 19-9 (CA19-9), in two groups of gynecologic cancer patients who had bilateral ovarian and bilateral fallopian excisions and were consuming either 0 or 50 mL of OO every morning. This study showed a significant decrease in the concentrations of CA125, CEA, and AFP in the OO consumption group [137]. OO in combination with vitamin D3, K1, and B6 also showed beneficial effects on platelet function and nitrosative stress prevention in healthy postmenopausal women [138]. Salvini et al. reported that high EVOO consumption, especially HT, prevented oxidative DNA damage in postmenopausal women [139]. Because of the limited number of patients, further studies are required.

Although the evidence is relatively limited, OO and its components have a positive impact on other aspects of women’s health, such as menstruation and sex. This study showed a similar effect of EVOO and ibuprofen in relieving the symptoms of primary dysmenorrhea, including pain scores and pain durations [140]. Sexual disorders are a common disorder in breast cancer survivors. Juraskova et al. showed that OO, during intercourse is one of the factors of OVERcome therapy, and like OO, vaginal exercise, and moisturizer, improved dyspareunia and sexual disorders in breast cancer patients [141].

## 5. Osteoporosis

Osteoporosis is a common chronic disease that affects most elderly persons, with women accounting for two-thirds of cases. The risk of osteoporosis increases dramatically in the postmenopausal period. Osteoporosis is a complication that leads to an increase in mortality in patients with osteoporosis [142]. Olive and olive polyphenols have been shown to increase bone mineral density and protect bone health [143]. Liu et al. reported that EVOO increased bone mineral density (BMD) in rats in an artificial menopause state due to ovariectomy [137]. Hagiwara et al. also reported the suppression of bone loss in ovariectomized mice when they used OLP and HT orally at 3-day intervals [144]. Puel et al. in several studies showed the effect of OO and its components in bone loss prevention in animal models [145,146,147,148]. Saleh et al. also showed similar results in osteoporosis models in rats [149]. Several studies have suggested that olive polyphenols protect bone health via oxidative stress reduction and anti-inflammatory effects. Olive polyphenols enhance the growth and differentiation of pre-osteoblasts and decrease osteoclast formation [143,144,150,151]. Gamma-linolenic acid originating from OO inhibits bone resorption and increases calcium levels in bone [152]. Filip et al. showed that polyphenol extract from OO increases osteocalcin concentration, a bone formation marker, and may help maintain lumbar BMD [153]. In contrast, Keiler et al. showed that using the total polyphenolic fraction of EVOO did not attenuate bone loss due to ovariectomy in rat models [154].

## 6. Cardiovascular Diseases and Type 2 Diabetes

OO positively impacts cardiovascular diseases. The available data demonstrated its protective role on vascular endothelial functions, lowering triglyceride levels, LDL-cholesterol reduction, pro-thrombotic reduction, and anti-atherogenic effects [155,156,157,158,159,160]. EVOO also improved dyslipidemia in postmenopausal women [161]. Jimenez-Morales showed that EVOO interacted with the NOS3 Glu298Asp polymorphism to reduce endothelial dysfunction in patients with metabolic syndrome [160]. The protective effect of OO was observed in a meta-analysis, and OO consumption can reduce the risk of coronary heart disease and stroke [162]. OO also has anti-inflammatory and antioxidant effects [163,164]. The beneficial effects of OO were observed in young women with mild hypertension. Additionally, OO enhanced endothelial function in this group [165]. Moreover, OO showed beneficial effects on anti-inflammatory markers related to cardiovascular diseases, such as C-reactive protein and interleukin-6 [166]. Lockyer et al. supported that OLE protects vascular function and that OLE also significantly decreases the concentration of the cytokine IL-8. This study used a digital volume pulse to measure vascular function in a randomized, double-blind, placebo-controlled, crossover, acute intervention trial in humans [167]. Filip et al. documented that polyphenol extract (Bonolive^®^) from olives decreased the total and LDL-cholesterol levels in postmenopausal women [153].

OO consumption also reduced the risk of type 2 diabetes in a meta-analysis study [168]. OLP also showed a potential effect in preventing hypoglycemia and oxidative stress-related complications in diabetic rabbits via a positive impact on enzymatic and non-enzymatic antioxidants [164].

## 7. Conclusions and Future Directions

The collected evidence showed the beneficial effects of OO on women’s health, especially in breast cancer, ovarian cancer, postmenopausal osteoporosis, cardiovascular disease, type 2 diabetes, and other disorders, along with the potential action mechanisms. Two groups of OO constituents were investigated: monounsaturated fatty acids (oleic acid) and the phenolic components. However, the bioactivities of the two groups might have contrasting effects, for instance, in cervical cancer. Almost all human studies have evaluated the effect of OO in the context of MD, so interpreting these studies might be challenging. However, evidence for gynecologic malignancy is limited, and the results remain inconsistent. Therefore, further studies are required to clarify the role of OO in this disease group, especially the active components, and to investigate the underlying mechanisms. The roles of OO in various aspects of women’s health are summarized in Table 1.

## Figures and Tables

**Table 1:** The role of OO in various aspects of women’s health.

Diseases	Products	Author	Study Design	Results
Osteoporosis	OOE	Casado-Diaz et al. 2017 [169]	Human mesenchymal stem cell and serum from postmenopausal women	Increased osteoblastogenesis
	Polyphenol extract from OO (Bonolive^®^)	Filip et al. 2015 [153]	A double blind, placebo-controlled study in 64 postmenopausal women	Increased osteocalcin levels
				Lumbar BMD maintenance compared to BMD reduction in the control group
	Total phenolic extract from EVOO	Keiler et al. 2013 [154]	Ovariectomized rats	No attenuation of bone loss
	OLP and HT	Hagiwara et al. 2011 [144]	MC3T3-E1 cell line, ovariectomized mice	Decreased bone loss in ovariectomized mice
	OO	Saleh et al. 2011 [149]	Ovariectomy-induced osteoporosis rats	Decline in bone loss
	Black lucques olives 2007	Puel et al. 2004, 2006, 2007, 2008, [145,146,147,148]	Ovariectomy/inflammation model	Increase in bone mineral density
	EVOO and OLP 2004			
	OLP 2006			
	HT and tyrosol 2008			
	Gamma-linolenic acid	Claassen et al. 1995 [152]	Rats	Inhibition of bone resorption
				Increase in calcium level
Postmenopausal disorders				
	OO plus Vitamin D3, K, B6	Vigini et al. 2017 [138]	Human, single-center, randomized placebo-controlled trial	Reduction in nitric oxide levels
				Maintenance of platelet function
	EVOO	Anderson-Vasquez et al. 2015 [161]	A prospective, longitudinal and comparative study, 18 healthy postmenopausal women	Dyslipidemia improvement
	Polyphenol extract from OO (Bonolive^®^_)_	Filip et al. 2015 [153]	A double blind, placebo-controlled study, 64 postmenopausal women	Decreased the total and LDL-cholesterol
	High-phenol EVOO	Salvini et al. 2006 [139]	Randomized cross-over intervention trial, postmenopausal women	Prevented oxidative DNA damage
Ovarian cancer	OLP	Sheikhshabani et al. 2021 [53]	A2780S and A2780/CP cell lines	Increased apoptosis
				inhibition of cell proliferation
				Decreases in cisplatin resistance
	OLE	Bennot-Dominguez et al. 2021 [52]	MDA-MB-231 and OVCAR-3	Viability inhibition, increased apoptosis, increased ROS production, mitochondria dysfunction was induced
	OLP	Xing et al. 2017 [55]	In vitro in the Caov3 and Skov3 cell line and in a xenograft mouse model	Upregulated miR-299 expression and inhibited HPSE1 expression
	Oleic acid	Menendez et al. [62]	SK-OV3	Repressed HER2-neu expression via PEA3 protein action
		Tzonou et al. 1993 [63]	Case-control	Risk reduction
	OO	Bosetti et al. 2002 [64]	Case-control	Risk reduction
	OO	Bosetti et al. 2009 [39]	Case-control	Risk reduction
Breast cancer	S-(−)-Oleocanthal (OC)	Qusa et al. 2021 [93]	MDA-MB-231 in vivo using two kinds of animal models: breast cancer patient-derived xenograft model and transgenic MMTV-PyVT	Inhibited cancer progression and metastasis. Investigated the mechanism at the gene level.
				Controlled the gene related to progression and metastasis
	OLP	Asgharzade et al. 2020 [79]	MCF-7 and MDA-MB-231	Inhibited cell proliferation
				Increased apoptosis
				Dysregulated miRNA
	OLP	Messeha et al. 2020 [82]	MDA-MB-468 and MDA-MB-231	MDA-MB-468 is more susceptible to OLP than MDA-MB-231
	OLP and HT	Lu et al. 2020 [90]	MCF7 and T47D	Decreased migration and invasion via autophagy activation
	OLA	Mazzei et al. 2020 [84]	MDA-MB-231, tamoxifen-resistant MCF-7	
	OLE	Benot-Dominguez et al. 2020 [52]	MDA-MB-231	Inhibited cell proliferation
				Induced apoptotic activity
				Increased ROS generation
	OLP	Reboredo-Rodríguez et al. 2018	MCF-7	Induced cell death and increased ROS production
	EVOO	Corominas-Faja et al. 2018 [112]	In vivo and in vitro HMLER, MCF10DCIS.com, SUM-159, MCF-7	Inhibited mammosphere formation, decreased tumor formation, regulated the expression of stem cell fates, inhibited self-renewal capacities via DNMT regulation and mTOR inhibition.
	OLP	Bayat et al. 2018 [81]	MCF-7	Induced apoptosis, decreased migration and invasion
				Decreased HDAC2 and HDAC3 expression
	OLP	Mansouri et al. 2018 [91]	MCF-7	Inhibited cell growth and invasion Induced apoptosis via HDAC regulation
	OLP	Choupani et al. 2018 [88]	MCF-7	Inhibited the migration via EMT repression by decreasing sirtuin1 expression
	OLP and HT	Chimento et al. 2014 [170]	ER-negative SKBR3	
	OLP	Hassan et al. 2013 [83]	MCF-7	P53 pathway activation
	OLP	Elamin et al. 2012 [85]	MDA-MB-231, MCF-7, MCF-10A	Delayed the cell cycle
				Decreased NF-kB and cyclin D-1 expression, p21 activation.
	OLP and HT	Odiatou et al. 2012 [171]	MDA-MB-231	Produced H_2_O_2_ led to DNA damage
				Decreased cell viability
	OLP	Hassan et al. 2012 [172]	MDA	Decreased MMP-2 and MMP-9 expression and increased TIMP1 and TIMP4 expression
	OLE	Fu et al. 2010 [173]	SKBR3, MCF-7, JIMT-1	Inhibit the cell proliferation
	HT	Bouallagui et al. 2010 [89]	MCF-7	Inhibited cell growth
				Cell cycle arrest (reduced expression of pin-1 resulted in decreased cyclinD1 expression)
	OLP and HT	Sirianni et al. 2010 [92]	MCF-7	Inhibited the activation of extracellular regulated kinase 1/2 that is dependent on E2
	OLP and Hydrotrosol	Han et al. 2009 [78]	MCF-7	Inhibited cell proliferation
				Induce cell apoptosis and G1 cell cycle arrest
	OLE	Goulas et al. 2008 [174]	MCF-7	Inhibited cell proliferation
	EVOO	Menendez et al. 2008 [100]	MCF-7 and SKBR3	Inhibited HER2 protein kinase activity
	EVOO	Menendez et al. 2008 [101]	MCF-7 and SKBR3	Inhibited the lipogenic enzyme expression in HER2-overexpression
	EVOO	Menendez et al. 2007 [77]	MCF-7 and SKBR3	Inhibited HER2
				Increases the effect of trastuzumab in SKBR3 and reversed the resistance to trastuzumab
	OO	Menendez et al. 2006 [62]	SK-Br3 and MDA_MB-231	Repressed HER2-neu expression via PEA3 protein action
	OO	Sealy et al. 2021 [116]	Meta-analysis	May reduce the risk but there was no significant relationship between the dose of OO and risk
	HT+omega-3 fatty acid+curcumin	Martinez et al. 2019 [114]	Clinical trial in early stage breast cancer patients using hormone	Reduced CRP
				Ameliorated pain
	OO	Xin et al. 2015 [47]	Meta-analysis	Reduced the risk
	OO	Pelucchi et al. 2011 [46]	Meta-analysis	Reduced the risk
	OO	Psaltopoulou et al. 2011 [45]	Systemic review and meta-analysis	Reduced the risk
	OO	Lipworth et al. 1997 [115]	Meta-analysis	Reduced the risk
Cervical cancer	EVOO	Toric et al. 2020 [120]	HeLa	Inhibited cell growth
	EVOO	Kouka et al. 2019 [121]	HeLa	Increased antioxidants
	Oleic acid	Zhang et al. 2019 [125]	HeLa	Increased cell proliferation, migration, and tumor growth
				Showed the different gene expression patterns altered by OO diet
	Oleic acid	Yang et al. 2018 [124]	HeLa	Enhanced tumor growth via CD31 induction by Scr?/ RK upregulation
	OLP	Yao et al. 2014 [123]	HeLa	Induced apoptosis via JNK/SPAK upregulation
Endometrial cancer				
	OO	Tzonou et al. 1996 [175]	Case control study	Reduced the risk
Vaginal and vulvar cancer			Not available	

## Abbreviations

PEA3	Polyomavirus enhancer activator 3
DNMT	DNA methyltransferase
EVOO-PE	EVOO-derived crude phenolic extract
CSC	cancer stem cell
DOLA	decarboxymethyl oleuropein aglycone
EMT	Epithelial-mesenchymal transition
IOC	International Olive Council
EVOO	extra virgin olive oil
OLE	olive leaf extract
OLP	oleuropein
OC	S-(-)-Oleocanthal
PA-1	plasminogen activator inhibitor-1
MT	Mediterranean
MD	Mediterranean diet
HT	hydroxytyrosol
OO	olive oil
ROS	reactive oxygen species
HDCA	histone deacetylase
MMP	matrix metalloproteinase
PTP1B	protein tyrosine phosphatase 1B
FASN	fatty acid synthetase
HPV	human papillomavirus
BMD	bone mineral density
